# Suppressing Colloidal Quantum Dot Multimer Fusion Leads to High‐Performance InSb Infrared Photodetectors

**DOI:** 10.1002/advs.202502775

**Published:** 2025-05-08

**Authors:** Lucheng Peng, Yongjie Wang, Carmelita Rodà, Aditya Malla, Miguel Dosil, Debranjan Mandal, Gerasimos Konstantatos

**Affiliations:** ^1^ ICFO‐Insitut de Ciencies Fotoniques The Barcelona Institute of Science and Technology Castelldefels Barcelona 08860 Spain; ^2^ ICREA‐Institució Catalana de Recerca i Estudiats Avançats Lluis Companys 23 Barcelona 08010 Spain

**Keywords:** band‐tail states, InSb, photodetectors, quantum dots, short‐wave infrared

## Abstract

Environmentally friendly InSb colloidal quantum dots (CQDs) short‐wave infrared (SWIR) photodetectors feature characteristics of low‐cost, high‐volume scalability, CMOS integrability, and compliance with RoHS regulations, and hold great commercial potential. Yet, their performance falls short of commercially relevant specifications. In this work, it is posited that CQD fusion observed in these dots leads to the formation of band‐tail trap states and it is further demonstrated that avoidance of such band‐tail trap states is crucial for device performance. By doing so, InSb CQDs SWIR photodetectors are reported with compelling performance metrics, including a dark current of 4 µA cm^−2^, EQE of ≈20% (at −1 V), a linear dynamic range over 140 dB and a response time of 90 ns. This represents a more than ten‐fold reduction in dark current compared to previously report InSb CQD photodetectors in the SWIR range. The record high PLQY of 10% for InSb/InP CQDs taken together with the high EQE of the device at zero bias confirm the achievement of high‐quality InSb CQDs through the suppression of band‐tail trap states and passivation of surface defects.

## Graphical abstract

This work shows that the fusion of InSb colloidal quantum dots (CQDs) leads to the formation of band‐tail trap states that are detrimental to the performance of optoelectronic devices. These results highlight the critical role of suppressing band‐tail trap states and passivating surface defects in enhancing device performance and motivate toward the development of highly monodisperse CQDs for high‐performance optoelectronic devices.

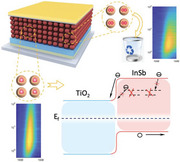

## 1 Introduction

Environmentally friendly colloidal quantum dot (CQD)‐based short‐wave infrared (SWIR) photodetectors would open up a huge number of applications, such as LIDAR and 3D imaging for automotive and Augmented/Virtual Reality (AR/VR),^[^ ^1^ ^]^ night vision for surveillance,^[^ ^2^ ^]^ spectroscopy for food quality inspection,^[^ ^3^, ^4^ ^]^ and bio‐imaging etc.,^[^ ^5^ ^]^ due to the low‐cost, high‐volume scalability, complementary metal‐oxide semiconductor (CMOS) integrability and compliance with RoHS (restriction of hazardous substances) regulations. Recently, efforts have been dedicated to developing heavy‐metal‐free CQDs materials and photodetectors in the SWIR region, such as indium arsenide (InAs),^[^ ^6^, ^7^, ^8^, ^9^, ^10^, ^11^, ^12^ ^]^ indium antimonide (InSb),^[^ ^13^, ^14^, ^15^, ^16^, ^17^, ^18^, ^19^ ^]^ and silver telluride (Ag_2_Te).^[^ ^20^ ^]^ Among these candidates, InSb CQDs stand out as one of the most promising alternatives due to the strong covalent bond character, and the low bulk bandgap allowing access to longer wavelengths within the quantum confinement regime.^[^ ^21^ ^]^ Despite some recent reports on the synthesis of InSb CQDs,^[^ ^14^, ^15^, ^22^ ^]^ and the realization of SWIR photodetectors,^[^ ^16^, ^17^, ^18^, ^19^ ^]^ the performance of InSb CQDs SWIR photodetectors lags behind practical application requirements, in terms of the dark current and external quantum efficiency (EQE).

Surface defects have been widely recognized as the main origin of trap‐states, a critical factor influencing the performance of CQD‐based optoelectronic devices. Prior efforts to improve the performance of InSb CQDs SWIR photodetectors have predominantly focused on passivating these trap states. Strategies include optimizing ligands during the ligand exchange process,^[^ ^16^, ^17^, ^18^ ^]^ refining precursors combination during synthesis,^[^ ^17^ ^]^ applying post‐synthesis Z‐type ligand treatments,^[^ ^19^ ^]^ and developing core–shell structures.^[^ ^19^ ^]^ While these approaches have led to performance improvements, they have largely overlooked the impact on device performance of band‐tail trap state formation in CQDs originating from undesired epitaxial fusion of CQDs.^[^ ^23^ ^]^ This issue is particularly significant for InSb CQDs due to their strong covalent bond character which is not favorable for the precise control of size and size distribution, thus increasing the risk of fusion of InSb CQDs due to the coalescence/attachment growth^[^ ^15^ ^]^ and leading to the formation of band‐tail trap states. Therefore, addressing concomitantly surface defects and band‐tail trap states may offer a more comprehensive pathway for enhancing device performance.

In this work, we first confirm that coalescence growth occurs during the synthesis of InSb CQDs, creating severe band‐tail trap states that reduce carrier lifetime, decrease PLQY, and increase the dark current of InSb CQDs SWIR photodetectors. By removing these fused CQDs, the high‐quality InSb CQDs with a sharp first exciton absorption peak and a peak‐to‐valley ratio of 1.65 is achieved. Surface defects are further passivated by the InSb/InP core–shell structures,^[^ ^19^ ^]^ achieving a record photoluminescence quantum yield (PLQY) of 10%. Leveraging these well‐passivated InSb/InP core–shell CQDs, we report InSb CQD SWIR photodetector which demonstrates the lowest dark current of 4 µA cm^−2^ at −1 V, EQE of 12% (0 V) and 21% (−1 V) at 1200 nm, and EQE of 9% (0 V) and 18% (−1 V) at 1350 nm, a linear dynamic range over 140 dB and a response time of 90 ns. We conclude that suppression of band‐tail trap states, along with surface passivation, are key for realizing high‐performance optoelectronic devices.

## 2 Results and Discussion

**Figure 1:**
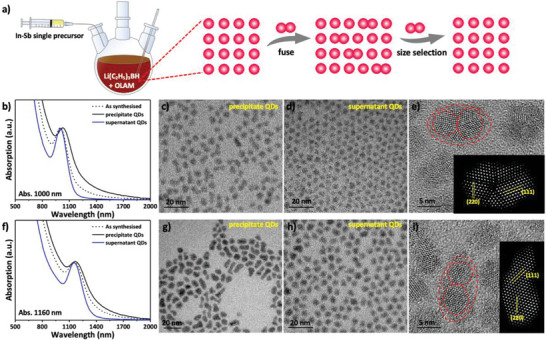
a) Schematic illustration of the synthesis of InSb CQDs via continuous precursor injection of “single‐source” precursor and the size selection process for the suppression of band‐tail trap states. b) The absorption spectra of 1000 nm InSb CQDs before and after size selection. c–e) The corresponding TEM and HRTEM images of 1000 nm InSb CQDs. f) The absorption spectra of 1160 nm InSb CQDs before and after size selection. g–i) The corresponding TEM and HRTEM images of 1160 nm InSb CQDs.

The currently obtained InSb CQDs almost universally exhibit poor size uniformity manifested by an exciton absorption peak with a low peak‐to‐valley ratio and the presence of an absorption tail.^[^ ^15^, ^16^, ^17^, ^22^ ^]^ As indicated by the Donega group, InSb CQDs prepared by the “single‐source” precursor hot‐injection method follow the coalescent growth mechanism^[^ ^15^ ^]^ and are likely to produce fused CQDs during the synthesis. How these fused CQDs affect the optical/electronic properties of the CQDs as well as the device performance has remained elusive. In our previous work, we adopted the “single‐source” precursor approach via a continuous precursor injection process instead of hot injection, which allowed us to obtain InSb CQDs with well‐distinct excitonic peaks.^[^ ^19^ ^]^ To carefully evaluate the presence of multimer CQDs during the growth process, we carried out the size selection process, as illustrated in **Figure** **1**a. The absorption spectra of InSb CQDs with an excitonic peak at 1000 nm before and after size selection were collected (Figure 1b). It can be clearly observed that the precipitate samples exhibit a dominated tail absorption (black line), whereas the supernatant samples exhibit a clean, flat below‐bandgap baseline and a well‐distinct first exciton peak with the peak‐to‐valley ratio of 1.65 (blue line), rendering them the most monodisperse InSb CQDs reported to date as shown in Table S1 (Supporting Information). To further evaluate the reproducibility of this synthesis and size‐selection method, we summarized ten different batches of InSb CQDs with a peak‐to‐valley ratio of 1.66 ± 0.03 (Figure S1, Supporting Information) indicating a low batch‐to‐batch variability. Transmission electron microscopy (TEM) images further revealed that the precipitate samples contained numerous fused CQDs with sizes ranging from ≈2.5 to 6 nm (Figure 1c,e), depending on the number of CQDs constituting the multimer. In contrast, the supernatant samples display a narrow size distribution, high monodispersity (polydispersity <10%, Figure S1, Supporting Information), and a size of ≈2.6 nm (Figure 1d). We performed the same processing for InSb CQDs with an absorption peak at 1160 nm, and both the absorption spectra (Figure 1f) and TEM images (Figure 1g–i) for the precipitate and supernatant samples demonstrated consistent patterns. Combining the absorption spectra and TEM characterizations, we confirmed that the tail absorption of InSb CQDs is caused by fused particles.

**Figure 2:**
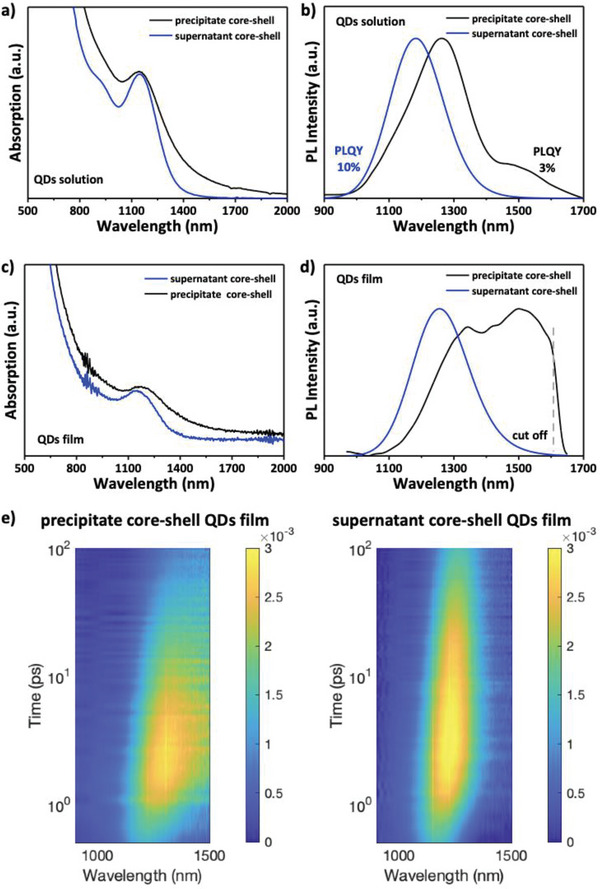
a, b) Absorption and PL spectra of the supernatant and precipitate InSb/InP core‐shell CQDs in solution. c, d) Absorption and PL spectra of the supernatant and precipitate InSb/InP core‐shell CQDs ligand‐exchanged films. e) Differential transmittance map ∆T/T of the precipitate and supernatant InSb/InP core‐shell films obtained excited the sample at 660 nm.

To better understand the impact of band‐tail absorption on the optical properties of these CQDs and to minimize the influence of surface defects, we performed InP shell growth on both the precipitate and supernatant CQDs. As shown in Figure S2 (Supporting Information), for both samples the absorbance shows a clear redshift compared to the starting cores due to the growth of the InP shell. Figures S3, S4 (Supporting Information) present the X‐ray diffraction (XRD) patterns and TEM images of the precipitate and supernatant CQDs before and after shell growth. Both core and core–shell samples reveal a pure cubic zinc blend crystal structure according to the XRD pattern. After the shell growth, the diffraction peaks shift to higher angles, and no additional diffraction peaks corresponding to InP were observed, indicating that a pure InSb/InP core–shell structure is achieved without any phase segregation. X‐ray photoelectron spectroscopy (XPS) measurement is conducted to analyze the surface. As shown in Figure S5 (Supporting Information), no signal at 539.9 eV, associated with Sb─O bonds, is detected in either the precipitate or supernatant core–shell CQDs, suggesting that the Sb dangling bonds on the surface are effectively passivated, consistent with our previous result.^[^ ^19^ ^]^ **Figure** **2**a,b show the absorption and PL spectra of the supernatant and precipitate core–shell CQDs in solution. Notably, the precipitate core–shell CQDs still exhibit pronounced band‐tail absorption. Accordingly, while the photoluminescence (PL) spectrum of the supernatant CQDs in solution consists of a symmetric narrow peak (FWHM 185 nm), the emission of the precipitated CQDs shows a long‐wavelength additional contribution to the spectrum in correspondence with the absorption tail, most likely ascribed to trap‐assisted recombination of charges in fused CQDs. In addition, the PLQY of the precipitate core–shell reaches up to 3%, in stark contrast to the record PLQY of 10% observed for the supernatant sample. This is likely due to the interface defects formed during the core fusion which cannot be well‐passivated by the grown InP shell. Additionally, this can also be due to carrier funneling to the larger CQD components of the fused multimers. To investigate the photo‐physics of the system under relevant conditions for device operation, transient absorption (TAS) and PL spectroscopy were carried out for both precipitate and supernatant CQDs in ligand‐exchanged solid‐state films. As shown in Figure 2c,d, the absorbance and PL spectra of the films are consistent with the data recorded in the solution, showing a well‐defined absorbance band‐edge tail and a broader PL spectrum for the precipitate core–shell film compared to the supernatant CQDs. Looking at the wavelength‐ and time‐resolved differential transmittance (∆T/T) in Figure 2e, a clear difference in the decay of the ground‐state bleach is observed when comparing precipitate and supernatant samples. Specifically, while the supernatant core–shell shows a narrow long‐lived bleach, the precipitate bleach is broader and decays faster. Notably, both samples show a progressive redshift of the bleach, which is absent in solution (Figure S6, Supporting Information) and can be ascribed to inter‐particle charge transfer due to the short exchange ligands. Hence, to quantify the decay time constants, the decay of the bleach integrated between 1000 and 1600 nm was considered. Accordingly, as shown in Figure S7 (Supporting Information), a double exponential fit of the decays lead to a time constant of 32±3 and 402±38 ps for the supernatant, values significantly longer than the precipitate (18±2 and 227±22 ps). Overall, this data highlights the beneficial effect of the size‐selection process on the optical properties of InSb/InP films.

**Figure 3:**
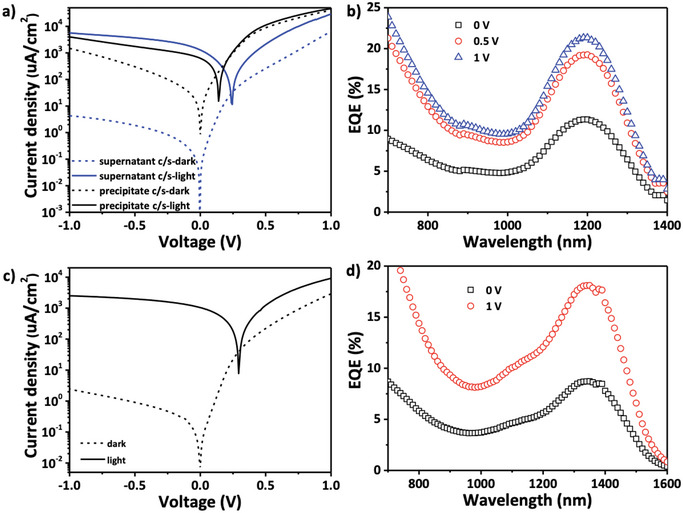
a) Current density‐voltage (*J–V*) curves of InSb/InP core‐shell CQDs photodetector in dark and illuminated conditions. b) External quantum efficiency (EQE) spectra of InSb/InP CQDs photodetector biased from 0–1V (reverse bias). c) Current density‐voltage (*J–V*) curves of larger InSb/InP core‐shell CQDs photodetector in dark and illuminated conditions. d) EQE spectra of larger InSb/InP CQDs photodetector with bias of 0 and 1V.

Ultraviolet photoelectron spectroscopy (UPS) is first carried out to measure the conduction band, valence band, and Fermi level of the InSb/InP core–shell CQDs film (Figure S8, Supporting Information). Based on this, the device architecture of indium‐tin‐oxide (ITO)/TiO_2_/InSb/PTAA/MoOx/Au and the corresponding band alignment are illustrated in Figure S9 (Supporting Information). The PTAA is doped by the hydrophobic Lewis acid dopant to increase the hole mobility.^[^ ^24^ ^]^ The current density–voltage (*J*–*V*) characteristic of the precipitate core–shell CQDs photodetector exhibits very poor rectification and significantly higher dark current density compared to the supernatant core–shell CQDs photodetector (**Figure** **3**a). This pronounced difference highlights the detrimental impact of band‐tail trap states in InSb CQDs on device performance. Notably, the supernatant core–shell CQDs photodiodes show a dark current density of ≈4 µA cm^−2^ at −1 V reverse bias, which is more than two orders of magnitude lower than previous reports,^[^ ^19^ ^]^ and currently is the record among all III‐V CQDs‐based SWIR photodetector fabricated by commercial, non‐pyrophoric precursors. Besides, we have checked the *J*–*V* curve based on the supernatant and precipitate InSb core CQDs devices (Figure S10, Supporting Information). The supernatant core also shows a lower dark current than the precipitate core, further supporting our claim. Both devices show extremely high dark current that is due to the influence of surface trap states of InSb core QDs according to our previous results.^[^ ^19^ ^]^ As shown in Figure 3b, the EQE of the supernatant core–shell CQDs photodetector is 12% at 1200 nm under 0 V bias, increasing to 21% under a reverse bias of −1 V, while the EQE of the photodetector made of precipitate core–shell CQDs is < 1% under 0 V bias (Figure S11, Supporting Information). Additionally, the same strategy is applied to larger InSb CQDs, with absorption spectra, XPS, and UPS analyses detailed in Figures S12, S13 (Supporting Information). The resulting photodetector achieves a dark current density of ≈3 µA cm^−2^ at −1 V reverse bias, along with EQE of 9% (0 V) and 18% (−1 V) at 1350 nm (Figure 3c,d).

**Figure 4:**
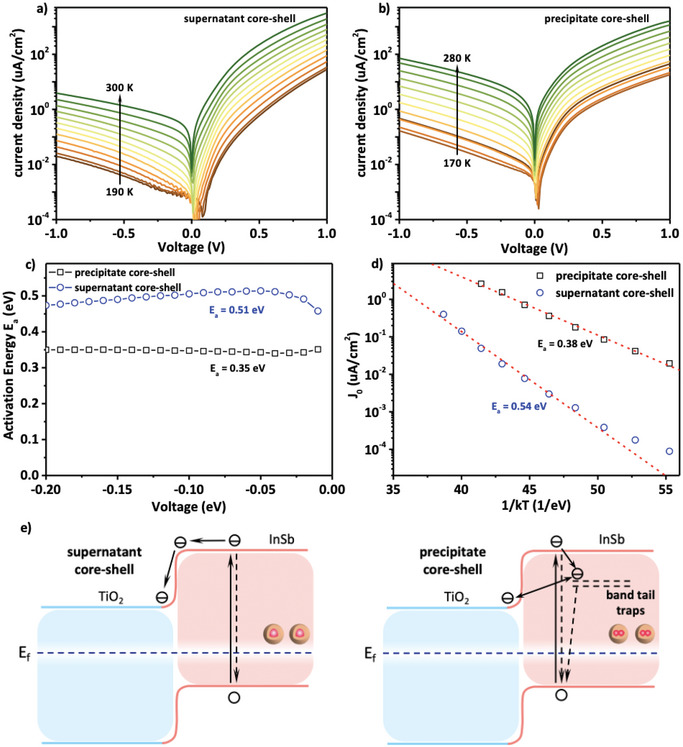
a) Temperature‐dependent dark current density‐voltage (*J–V–T*) curves of supernatant InSb/InP core‐shell CQDs photodetector. b) Temperature‐dependent dark current density‐voltage (*J–V–T*) curves of precipitate InSb/InP core‐shell CQDs photodetector. c) Activation energy of the supernatant InSb/InP core‐shell and precipitate InSb/InP core‐shell CQDs photodetector extracted from the corresponding *J–V–T* measurements. d) Fitting of reverse saturation dark current density with thermal generation model. e) The carrier generation and extraction process of the device based on the supernatant and precipitate InSb/InP core‐shell CQDs.

To further investigate the effects of band‐tail trap states on diode quality and dark current, temperature‐dependent current–density voltage (*J*–*V*) measurements are conducted based on the supernatant and precipitate core–shell CQDs. Both devices show diode‐like behavior and a clear reduction of reverse dark current with decreasing temperature (**Figure** **4**a,b), indicating a thermally activated process dominating dark current. Analysis of the temperature‐dependent *J*–*V* curves reveals activation energies of 0.51 eV for the supernatant core–shell CQDs photodiode and 0.35 eV for the precipitate core–shell CQDs photodiode (Figure 4c). Further investigation (activation energy calculation in Experimental Section) shows that the reverse dark current in both devices is dominated by thermally generated carriers under low reverse bias and by trap‐assisted carrier injection at higher reverse bias. Specifically, the reverse dark current in both devices is dominated by thermally generated carriers under low reverse bias. With the reverse bias increasing, the contribution from carrier injection current increases due to bias‐dependent carrier injection and becomes dominant under high reverse bias (> 0.5V). Thermally activated barriers of 0.54 and 0.38 eV for the supernatant and precipitate devices, respectively, are calculated from diode currents (Figure 4d; Figures S14, S15, Supporting Information), matching well with the calculated activation energy from dark JV curves at low reverse bias range. Furthermore, the extinction coefficient measurement is carried out to evaluate the absorption band edge of the supernatant and precipitate core–shell CQDs films. As shown in Figure S16 (Supporting Information), the precipitate core–shell CQDs film shows lower absorption onset compared to the supernatant core–shell CQDs film. These results indicate that band‐tail trap states reduce the effective bandgap, thereby increasing the dark current (detailed explanation in Experimental Section).^[^ ^26^, ^27^ ^]^ Figure 4e illustrates the difference in thermal carrier generation and carrier extraction process taking place in supernatant‐ and precipitated‐core–shell CQDs respectively: The interface defects formed by the fusion in precipitated core–shell CQDs, by inducing band‐tail ingap states thereby effectively reducing the material bandgap, can significantly increase the dark current and suppress the EQE of the device, thus resulting in a poor device performance.

**Figure 5:**
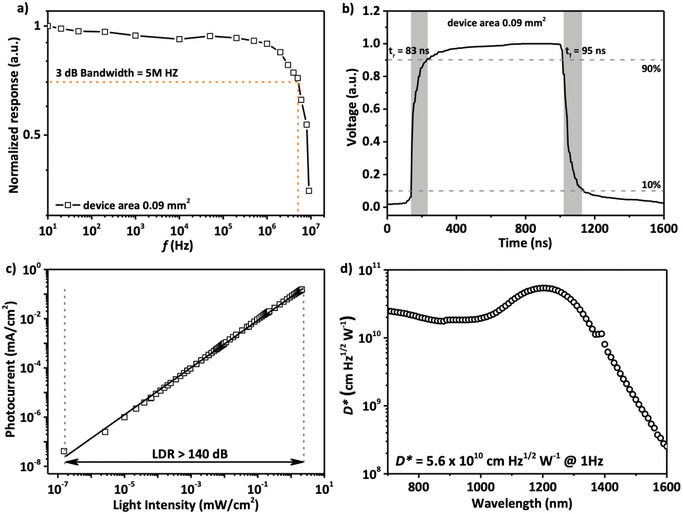
a) Frequency response bandwidth of InSb/InP core‐shell CQDs photodetector with a device area of 0.09 mm^2^. b) Response time of InSb/InP core‐shell CQDs photodetector with device area of 0.09 mm^2^. c) Linear dynamic range (LDR) of InSb/InP core‐shell CQDs photodetector under 1310 nm pulse light illumination. d) Specific detectivity spectrum of InSb/InP core‐shell CQDs photodetector at 1 Hz.

Next, we further characterized the photodiode performance based on supernatant core–shell CQDs. As shown in **Figure** **5**a, the 3 dB bandwidth is ≈5 MHz with the device area of 0.09 mm^2^. The transient photocurrent (TPC) measurement reveals a response time of 90 ns (Figure 5b), in accordance with the 3 dB bandwidth measurement. The linear dynamic range (LDR) of the device is measured by varying the incident light intensity. As shown in Figure 5c, the photocurrent‐maintained linearity with incident light power over a broad range and the LDR was estimated to be over 140 dB. The frequency‐dependent current noise spectrum of the device is measured by the transient‐current fast Fourier‐Transform (FFT) method (Figure S17, Supporting Information). The obtained noise spectrum shows a 1/f noise dominating at low frequency and reaches a flat noise floor of ≈3.6 × 10^−13^ A Hz^−0.5^ at a frequency of 1 Hz. Therefore, we can report room‐temperature specific detectivity of the device of D^*^ ≈ 5.6 × 10^10^ Jones at 1 Hz at 1200 nm (Figure 5d), which is 5 times higher than previous reports.^[^ ^19^ ^]^ The same measurement and calculation are applied to larger InSb CQDs, showing a maximum D^*^ ≈ 4.7 × 10^10^ Jones at 1380 nm at 1 Hz (Figure S18, Supporting Information), and the frequency‐dependent D^*^ is also provided in Figure S19 (Supporting Information). This specific detectivity represents a record of III‐V CQDs SWIR photodetectors in the low‐frequency regime which is of paramount importance for practical applications and particularly image sensors. We also checked the device stability under operation in the ambient environment (without any encapsulation); the device did not show any degradation after the overnight test (Figure S20, Supporting Information) which is consistent with our previous result.^[^ ^19^ ^]^ Last, in Figure S21 (Supporting Information), we summarize the EQE and dark current of this work and other III‐V CQDs photodetectors that are fabricated by commercial non‐pyrophoric precursors in the SWIR range at 1350 nm, which is a practically relevant wavelength regime for several applications.

## 3 Conclusion

In summary, we have identified that band‐tail trap states in InSb CQDs, originating from fused CQDs, reduce carrier lifetime and increase the dark current of the device, as evidenced by TAS analysis and temperature‐dependent *J*–*V* measurements. By eliminating these fused CQDs, high‐quality InSb CQDs with a sharp first exciton absorption peak and a peak‐to‐valley ratio of 1.65 are obtained. Surface defects were further passivated using InSb/InP core–shell structures, achieving a record PLQY of 10%. Utilizing these well‐passivated CQDs, we have reported InSb CQD SWIR photodetectors with an over an order of magnitude reduced dark current and a resultant increase in D^*^‐at conditions relevant for imaging applications by a factor of five over the previous reports. These results highlight the critical role of suppressing band‐tail trap states and passivating surface defects in enhancing device performance. We further showcase that besides chemical surface passivation, having high‐quality monodisperse nanocrystals plays an equally important role and our work further motivates the development of highly monodisperse III‐V colloidal quantum dots for high‐performance optoelectronic devices.

## 4 Experimental Section

### Chemicals

Oleylamine (OLA, 80%–90%) and Tris(trimethylsily)phosphine ((TMS)_3_P, 98%) were purchased from Thermal Scientific. Tris(dimethylamido)antimony‐(III) (Sb[NMe_2_]_3_, 99.99%) and indium(III) chloride (InCl_3_, 99.999%) were purchased from Strem Chemicals. Indium acetate (In(OAc)_3_, 99.99%), indium(III) iodide (InI_3_, 99.998%), 1‐octadecene (ODE, 90%), lithium triethylborohydride (LiEt_3_BH, Super‐Hydride, 1 m in tetrahydrofuran, THF), dioctyl ether (DOE, 99%), oleic acid (OA, 90%), Tris(pentafluorophenyl)borane (BCF, 95%), methanol (anhydrous, 99.8%) and toluene (anhydrous, 99.8%) were purchased from Sigma–Aldrich. Acetone (anhydrous, 98%) was purchased from Scharlab. Poly‐[bis(4‐phenyl)(2,4,6‐trimethylphenyl)]amine (PTAA) was purchased from Ossila. All chemicals were used as received except for oleylamine and oleic acid, which were degassed before use. The degassing was performed at 100 °C under reduced pressure (≈1 mbar) for 4 h.

### Preparation of Superhydride Solution

LiEt_3_BH (1.0 m) dissolved in tetrahydrofuran (THF) was used as purchased. A 100 mL amount of the superhydride solution was added to 50 mL of degassed dioctyl ether (DOE) and then evacuated on the Schlenk line for >4 h, until the THF was completely removed. **Caution**: LiEt_3_BH is very reactive in air and should be handled using air‐free processes. The final superhydride solution had a concentration of 2.0 m in DOE.

### Preparation of the In‐Sb Precursor Solution

1.6 mL toluene, 400 µL of OLA (1.2 mmol), and 115 µL of Sb[NMe_2_]_3_ were mixed together in a glovebox under N_2_ (H_2_O and O_2_ < 0.1 ppm) yielding a bright yellow solution. Then 0.6 mmol InCl_3_ was added and dissolved under stirring at room temperature for 5 min.

### Preparation of 0.2 m (TMS)_3_P‐ODE Solution

1 mL (TMS)_3_P solution was mixed with 16 mL degassed ODE, and the 0.2 m (TMS)_3_P‐ODE solution was formed.

### Synthesis of InSb Colloidal Quantum Dots

In a typical synthesis, 20 mL of degassed OLA was heated to 230 °C in a round‐bottom flask under constant stirring and N_2_. At this temperature, 4.5 mL of LiEt_3_BH in DOE (2.0 m, 9 mmol) was added in a dropwise manner within 2 min. During the addition, OLA acquired an orange color. Subsequently, 2 mL of the In‐Sb precursor solution was pumped into the organic mixture with the pumping rate of 1 mL min^−1^. After that, the temperature was kept at 230 °C for another 5 min and cooled down to room temperature naturally.

### InCl_3_ Post‐Treatment for InSb Colloidal Quantum Dots

Before the purification of InSb CQDs, 1.2 mmol InCl_3_ was loaded in a round‐bottom flask filled with N_2_, and then the as‐synthesized InSb CQDs crude solution was transferred into the flask and heated to 200 °C from room temperature for 30 min for the post‐treatment.

### Purification and Size‐Selection of InSb Colloidal Quantum Dots

After the post‐treatment, the reaction solution was transferred into the plastic centrifuge tubes inside the glovebox. The reaction mixture was separated in two portions, oleic acid (2 mL) was added to each and rotated for 2 min. Then 24 mL of MeOH was added, leading to turbidity. Centrifugation for 10 min at 6000 rpm yielded a black precipitate and yellowish supernatant. The yellowish supernatant was discarded, and the black precipitate was redispersed in 10 mL of toluene, and centrifugation for more than 10 min at 6000 rpm. The precipitate was discarded, then 2 mL of oleic acid was added to the supernatant, and 15 mL of MeOH was added. Again, centrifugation for 10 min at 6000 rpm yielded a black precipitate and colorless supernatant. The colorless supernatant was discarded, and the black precipitate was redispersed in 4 mL of anhydrous toluene. For the size‐selection process, 4 mL of acetone was added into the quantum dots solution in toluene, and centrifugation for 10 min at 6000 rpm. The precipitate was collected and redispersed in anhydrous toluene in the label of precipitate CQDs with a concentration of 50 mg mL^−1^. The supernatant was again added with another 8 mL of acetone and centrifugation for 10 min at 6000 rpm. The precipitate was collected and redispersed in anhydrous toluene in the label of supernatant CQDs with a concentration of 50 mg mL^−1^.

### Synthesis of InSb/InP Core–Shell Colloidal Quantum Dots

In a typical synthesis, 0.1 mmol In(OAc)_3_, 0.3 mmol OA, and 10 mL octadecene were mixed in a 25 mL flask and evacuated on the Schlenk line at 100 °C for 2h. Then the temperature was raised to 150 °C for the complete dissolving of In(OAc)_3_. Next, the ODE solution was cooled down to 60 °C and 1 mL 50 mg mL^−1^ InSb CQDs was added. After the addition of InSb CQDs, the solution was bubbled with N_2_ for 30 min at 60 °C. Then 400 µL (TMS)_3_P‐ODE solution (0.2 m) was added and the reaction temperature was raised to 270 °C for the growth of the InP shell. After 30 min growth of the InP shell, the reaction mixture was cooled down to room temperature naturally.

### Purification of InSb/InP Core–Shell Colloidal Quantum Dots

The reaction mixture was transferred into the plastic centrifuge tubes inside the glovebox and centrifuged for 10 min at 6000 rpm. The precipitate was discarded and 30 mL of acetone was added to the supernatant. Centrifugation for 10 min at 6000 rpm yielded a black precipitate and colorless supernatant. The colorless supernatant was discarded, the black precipitate was redispersed in 10 mL of toluene, and repeated the wash process. The final black precipitate was redispersed in anhydrous toluene, resulting in a 50 mg mL^−1^ InSb/InP CQDs solution for further characterization and device fabrication. All the synthesis and purification steps were carried out in an inert atmosphere under anhydrous conditions (N_2_ glovebox, H_2_O, and O_2_ < 0.1 ppm).

### Characterization of the Quantum Dots

UV–vis absorption measurements were performed with a Cary 5000 UV–vis–NIR spectroscope in solution. The absorption measurements of films were carried out using a Lambda 1050+ spectrometer with an integrating sphere accessory. The absorbance has been determined by measuring reflectance and transmittance configuration. For photoluminescence (PL) measurements, a four‐channel Thorlabs laser was used as excitation light, and a Kymera 328i spectrograph (Oxford Instruments, Andor) was used as the detector (<1600 nm). For the PLQY measurement, a 935 nm laser, an integration sphere, and the Andor detector were used. The XRD data were collected using a Rigaku SmartLab powder diffractometer in the Bragg–Brentano geometry with Cu Kα radiation on drop‐casted powder samples. TEM was performed at the Scientific and Technological Centres of the University of Barcelona. The TEM images were obtained using a JEOL 2100F microscope operating at an accelerating voltage of 200 kV. TEM samples were prepared by dropping diluted CQD solution on ultrathin carbon grids. The XPS/UPS measurements were performed with a SPECS PHOIBOS 150 hemispherical analyser under ultrahigh‐vacuum conditions (10^−10^ mbar) with a monochromatic Kα X‐ray source (1,486.74 eV) at the Institut Català de Nanociència i Nanotecnologia. The extinction coefficient was measured at various angles using a broadband Sopra Ellipsometer GES5E instrument. The SEMILAB spectroscopic ellipsometry analyzer software was utilized to fit a model of stacked layers of appropriate optical constants. Transient absorption spectroscopy was carried out using a femtosecond transient absorption spectrometer (Helios, Ultrafast System). Samples were excited using 110 fs‐pulses at 660 nm generated by the amplified 800 nm of a Ti:Sapphire laser (ACE Solstice, Spectra‐Physics, 1 kHz) through nonlinear frequency mixing in an optical parametric amplifier (Apollo‐T, Ultrafast Systems). Equally short probe beams were created using a non‐linear crystal covering the 750–1600 nm range. Pump pulses were delayed relative to the probe using a delay stage with a maximum delay of 7 ns.

### Device Fabrication

ITO‐covered glass substrates (Universität Stuttgart, Institut für Großflächige Mikroelektronik) were cleaned by sonication in soapy water, acetone, and isopropanol for 20 min each and dried with nitrogen, followed by 30 min UV/ozone treatment. TiO_2_ electron transport layer was then deposited with a sputtering technique to achieve a thickness of ≈40 nm. Three layers of InSb/InP CQDs were further spin‐coated from 50 mg mL^−1^ toluene solution via the layer‐by‐layer method. For each InSb/InP CQDs layer, one drop of InSb/InP CQDs solution was spin‐coated onto TiO_2_/ITO substrates during spinning (2,000 r.p.m.). Then, 5 mg mL^−1^ InI_3_/Methanol solution was applied to the CQDs film for 30 s, followed by two rinses–spin steps with methanol and once with toluene. Then 2 mg mL^−1^ PTAA with 5% BCF doping solution in toluene was spin‐coated onto InSb/InP CQDs film at 2000 rpm for 30 s. Finally, a Kurt J. Lesker NANO 36 system was used to deposit 10 nm MoO_x_ as the electronic blocking layer and 100 nm Au as the top electrode with a device area of 3.1 mm^2^. The device with a smaller device area was deposited with the special shadow mask.

### Device Characterization

All the device characterizations were performed in air under ambient conditions. Current–voltage (*I*–*V*) measurements were performed with a Keysight Semiconductor Parameter Analyzer (B1500A) with the devices kept in a shield box. The EQE was measured using a Newport Cornerstone 260 monochromator, a Thorlabs MC2000 chopper, a Stanford Research SR570 trans‐impedance amplifier, and a Stanford Research SR830 lock‐in amplifier. Calibrated Newport 818‐UV and 818‐IR photodetectors were used as the reference. To measure the 3dB bandwidth, a nanosecond laser (520 nm) was used as the incident light, which was modulated by a waveform generator (Agilent 33220A) at various frequencies with a 50% duty cycle. The output current was recorded with an oscilloscope. For linear dynamic range measurements, a four‐channel laser (Thorlabs) at 1310 nm was used as a light source at a frequency of 7 Hz modulated by an Agilent waveform generator. The light was directed by a beam‐splitter to a Newport 818‐IG detector and the device. The current was then measured by a semiconductor parameter analyzer. For the noise measurements, the frequency‐dependent current noise spectrum was measured at low frequency by the transient‐current fast Fourier‐Transform (FFT) method. The measured room‐temperature specific detectivity (D^*^) was calculated according to:

(1) $$D^{*}\;=\;\frac{R\sqrt{A\Delta f}}{i_{n}}$$

where D^*^ is expressed in units of cm Hz^1/2^ W^−1^ or Jones, A is the active area of the photodetector, *i*_n_ is the noise current spectral density, and Δ*f* is the noise bandwidth, here being 1 Hz. For the calculation of Figure 5d, where *i*_n_ is 3.6 × 10^−13^ A Hz^−0.5^ at 1 Hz, A is 0.0314 cm^2^ and R is 0.12 A W^−1^ at 1200 nm, hence the D* is 5.6 × 10^10^ Jones at 1200 nm at 1 Hz.

### Activation Energy Calculation

The temperature‐dependent dark current density–voltage (*JVT*) curves were first fitted with the equation to extract the overall activation energy:

(2) $$J_{d}\propto \exp\left(\frac{E_{a}}{k_{B}T}\right)$$

where *J*_d_ is the dark current density, *k_B_* is the Boltzmann constant, *T* is the temperature and *E_a_* is the activation energy. The *E_a_* was found to be 0.51 eV for the supernatant core–shell CQDs device and 0.35 eV for the precipitate core–shell CQDs device (Figure 4c).

In order to further separate the diode currents from leakage currents, the *JVT* curves were further fitted into an equation:^[^ ^25^ ^]^

(3) $$J_{d}=J_{0}\left[\exp\left(\frac{q\left(V-R_{s}J_{d}\right)}{nk_{B}T}-1\right)\right]+\frac{V-R_{s}J_{d}}{R_{p}}+k{\left(V-R_{s}J_{d}\right)}^{m}$$

where *J*_0_ is the reverse saturation current density of the diode, *n* is the ideality factor, *q* is the elementary charge, *V* is the voltage bias, *R_s_* is the series resistance, *R_p_* is the shunt resistance, *k* and *m* are constants related to non‐ohmic leakage current. Non‐Ohmic leakage can be attributed to mainly trap‐assisted tunneling current and interface defects.

We further fitted reverse saturation current density into the thermally activated generation model:

(4) $$J_{0}=J_{00}\;\exp\left(\frac{qE_{a}}{k_{B}T}\right)$$

where *J*_00_ is the prefector, *q* is the elementary charge, *k_B_* is the Boltzmann constant, *T* is the temperature and *E_a_* is the activation energy.

### Radiative Recombination Dark Current

According to detailed balance,^[^ ^26^, ^27^ ^]^ the radiative recombination dark current can be calculated as:

(5) $$J_{rad}=q\int A\left(E\right)\times \Phi_{BB}\left(E\right)dE$$

where q is the elemental charge, A(E) is the total absorption of the QD films, which is equivalent to the extinction coefficient, Φ_*BB*_(*E*) is the black body radiation, which peaks at ≈10 um at room temperature. The more overlapping between A(E) and Φ_*BB*_(*E*) leads to a larger integral and thus a higher dark current, which is the case in precipitate devices.

## Conflict of Interest

G.K. serves as co‐founder, shareholder, and scientific advisor at Qurv. The remaining authors declare no competing interests.

## Supporting information



Supporting Information

## Data Availability

The data that support the findings of this study are available from the corresponding author upon reasonable request.
