# Supplementary material for: Suppressing Colloidal Quantum Dot Multimer Fusion Leads to High‐Performance InSb Infrared Photodetectors
Source: Adv Sci (Weinh). 2025 May 8;12(27):2502775. doi: 10.1002/advs.202502775 (PMC12279176; doi:10.1002/advs.202502775)
Supplement: Supplementary file 1 — Supporting Information [file ADVS-12-2502775-s001.docx]

Supporting Information

Suppressing colloidal quantum dot multimer fusion leads to high performance InSb infrared photodetectors

Lucheng Peng*, Yongjie Wang, Carmelita Rodà, Aditya Malla, Miguel Dosil, Debranjan Mandal, and Gerasimos Konstantatos*

L. Peng, Y. Wang, C. Rodà, A. Malla, M. Dosil1, D. Mandal, G. Konstantatos

ICFO-Insitut de Ciencies Fotoniques, The Barcelona Institute of Science and Technology, Castelldefels, 08860 Barcelona, Spain.

G. Konstantatos

ICREA-Institució Catalana de Recerca i Estudiats Avançats, Lluis Companys 23, 08010 Barcelona, Spain.

E-mail: [gerasimos.konstantatos@icfo.eu](mailto:gerasimos.konstantatos@icfo.eu), [lucheng.peng@icfo.eu](mailto:lucheng.peng@icfo.eu)

**Table S1.** Comparison of our work and state-of-the-art InSb CQDs in terms of the peak to valley ratio of first exciton absorption peak.


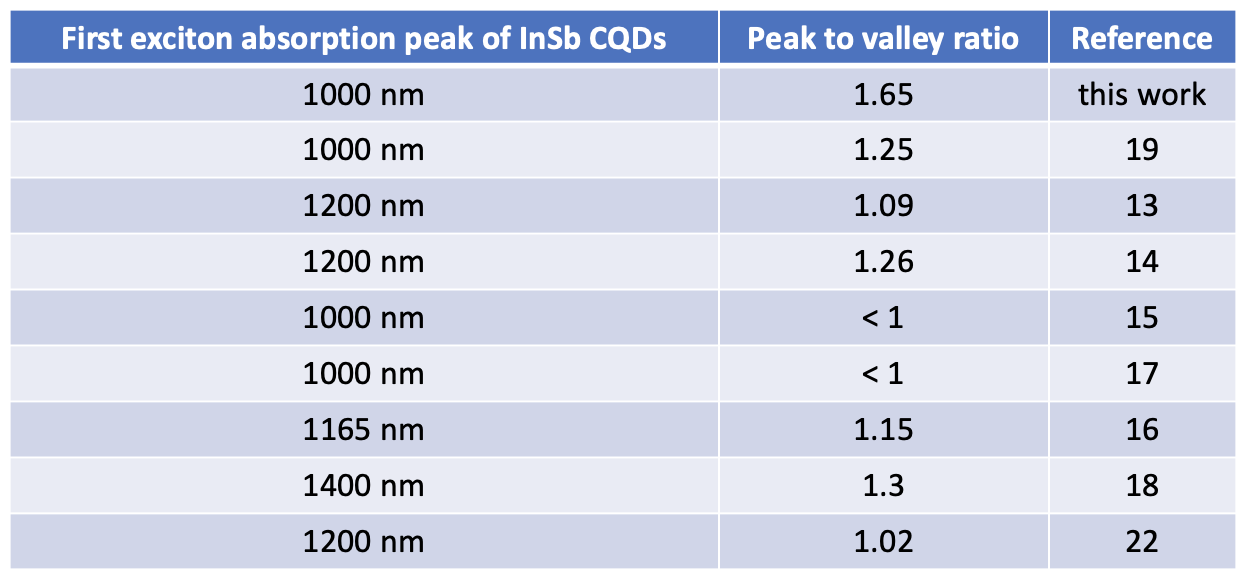


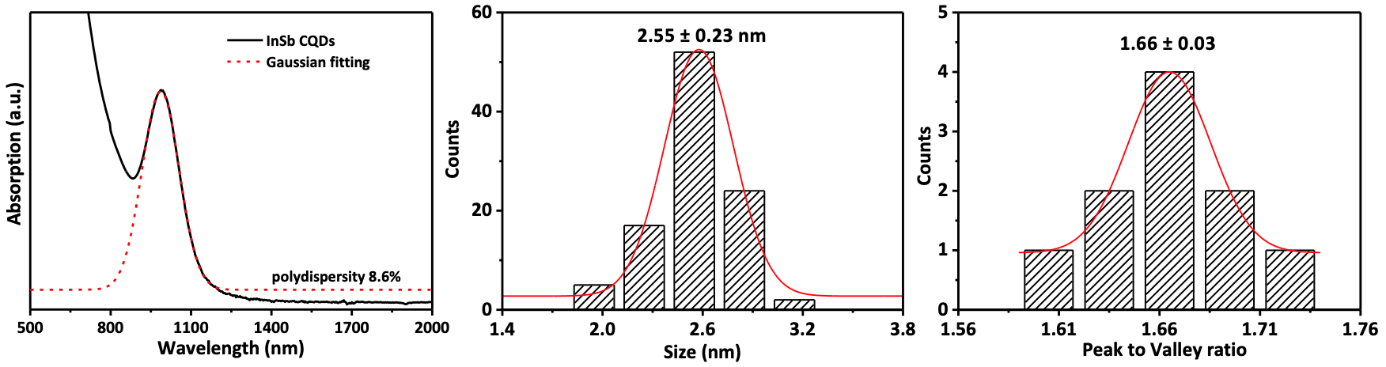


**Figure S1.** Size distribution histograms of the supernatant InSb CQDs according to the absorption spectrum (left) and TEM image (middle). The peak-to-valley ratio of ten different batches of supernatant InSb CQDs (right).


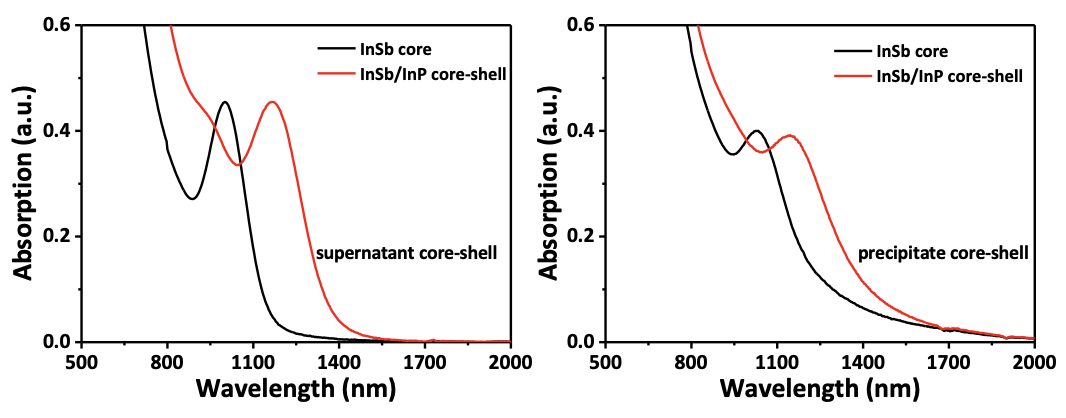


**Figure S2.** Absorption spectra of the supernatant and precipitate InSb CQDs before and after the InP shell growth.


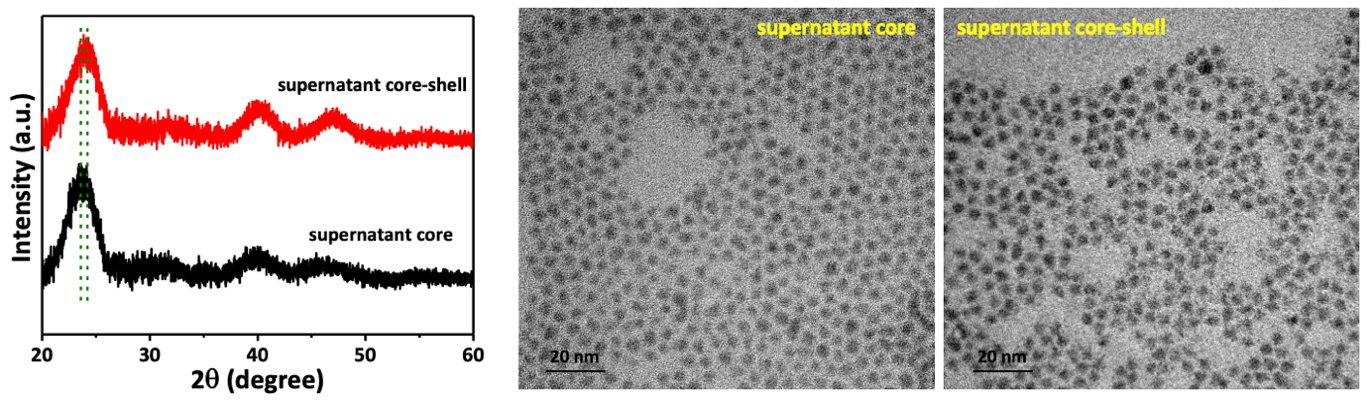


**Figure S3.** Powder X-ray diffraction (XRD) patterns and transmission electron microscopy (TEM) images of the supernatant InSb CQDs before and after the and InP shell growth. The diffraction peak of InSb core at 23.8^o^ moved to high angle at 24.3^o^ after the InP shell growth.


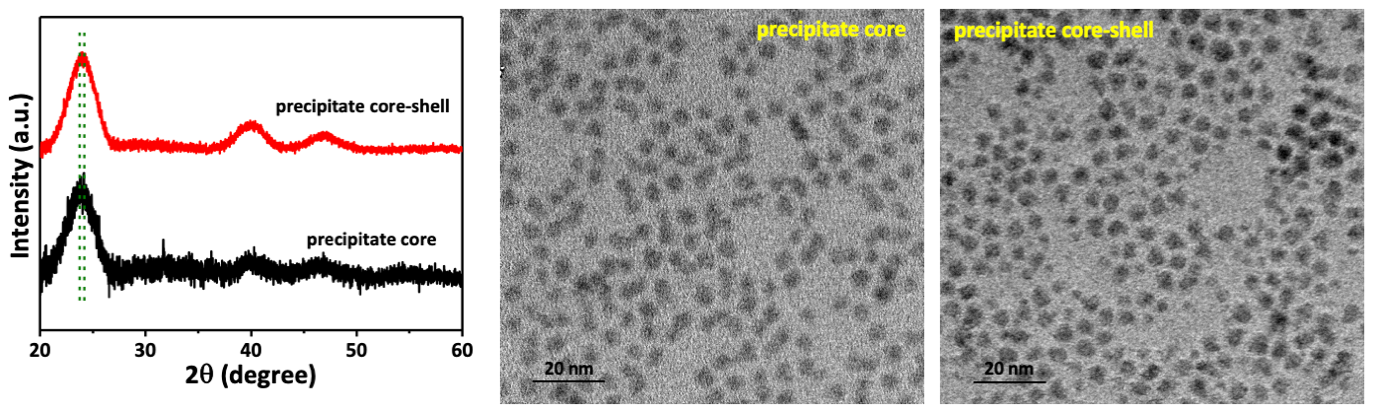


**Figure S4.** Powder X-ray diffraction (XRD) patterns and transmission electron microscopy (TEM) images of the precipitate InSb CQDs before and after the and InP shell growth.


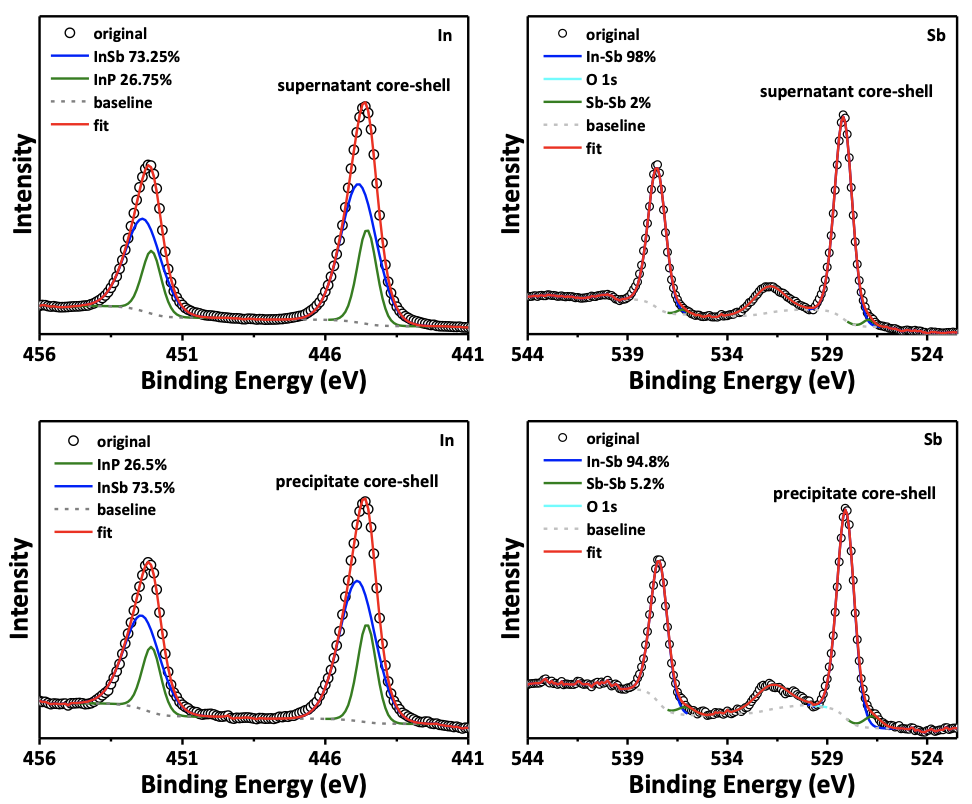


**Figure S5.** The In and Sb X-ray photoelectron spectroscopy (XPS) analysis of the supernatant and precipitate InSb/InP core-shell CQDs films. There is no signal at 539.9 eV, associated with Sb-O bonds, detected in either the precipitate or supernatant core-shell CQDs films.


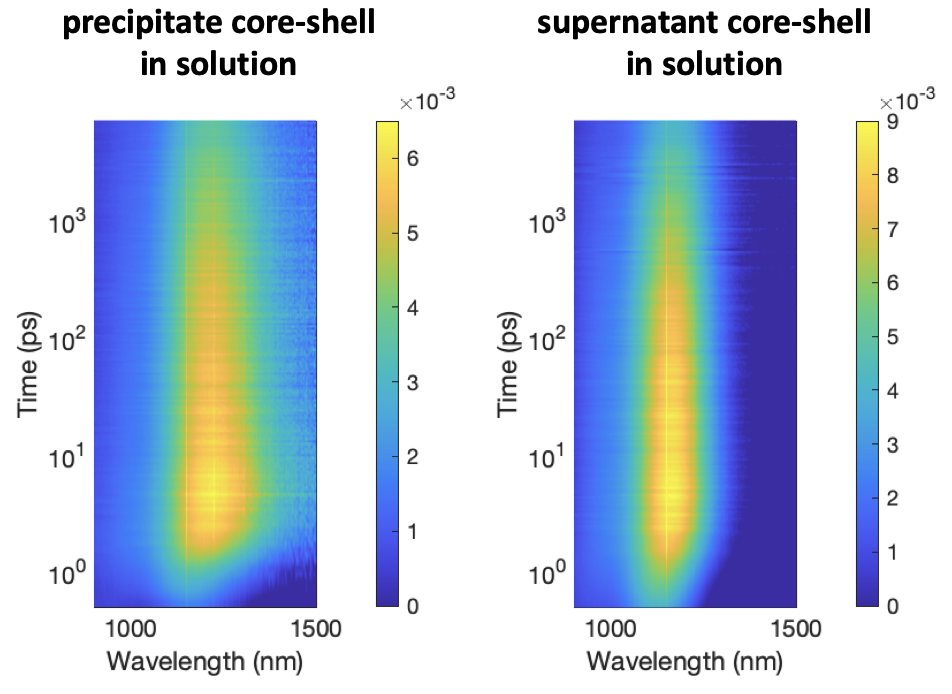


**Figure S6.** Differential transmittance map ∆T/T of the precipitate and supernatant InSb/InP core-shell CQDs in toluene obtained exciting the sample at 660 nm.


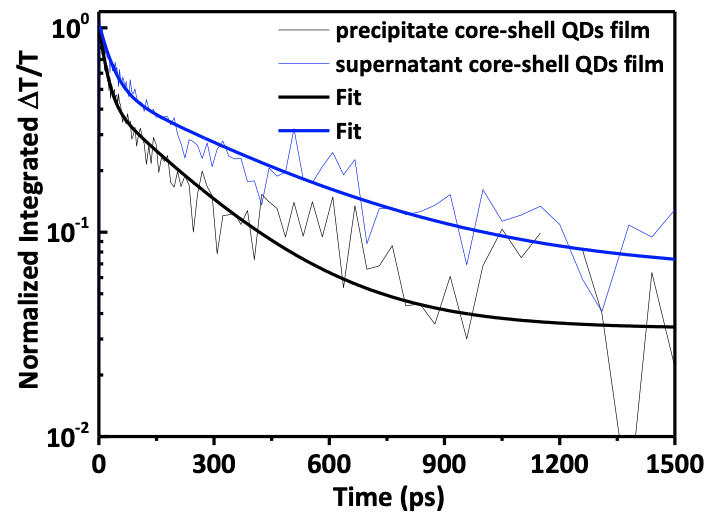


**Figure S7.** The normalized integrated transient absorption bleach of precipitate and supernatant InSb/InP core-shell CQDs films obtained exciting the sample at 660 nm.


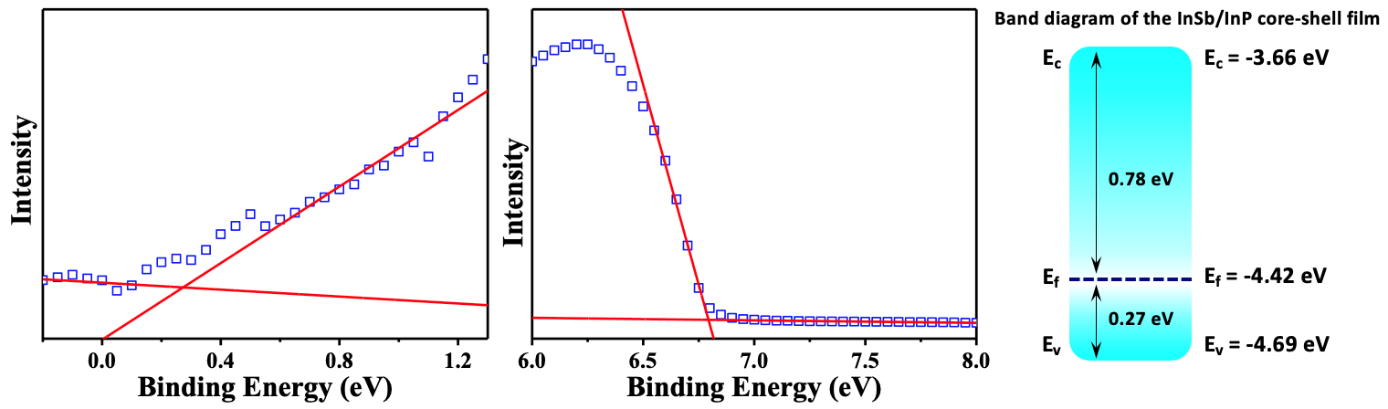


**Figure S8.** The energy level of the InSb/InP core-shell CQDs film analyzed by the UPS spectra after InI_3_ ligand exchange.


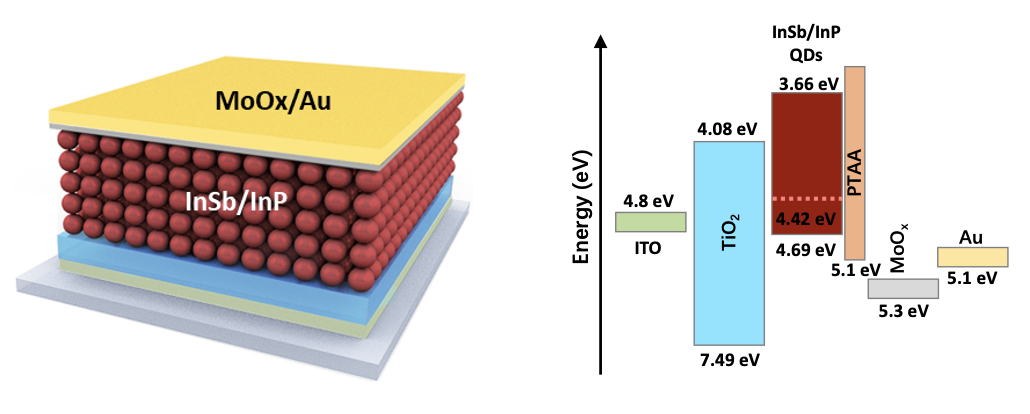


**Figure S9.** Schematics of InSb/InP core-shell CQDs SWIR photodetector (left). Band diagram of InSb/InP CQDs photodetector (right).


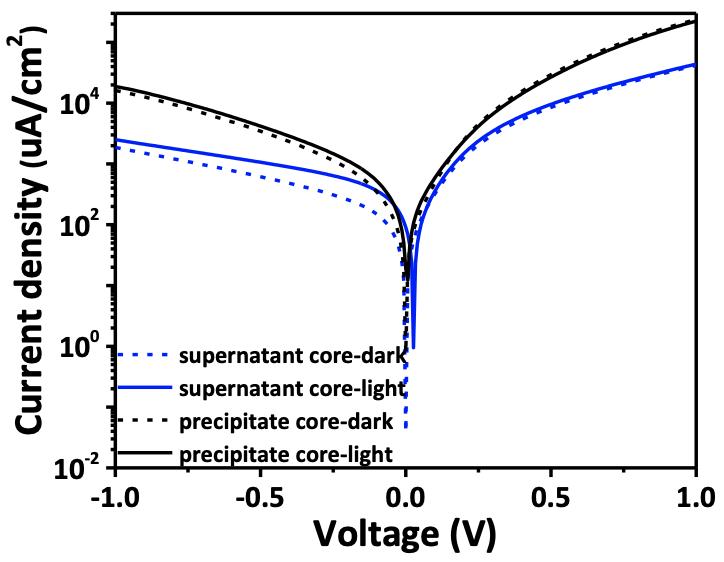


**Figure S10.** Current density-voltage (*J-V*) curves of InSb core CQDs photodetector in dark and illuminated condition.


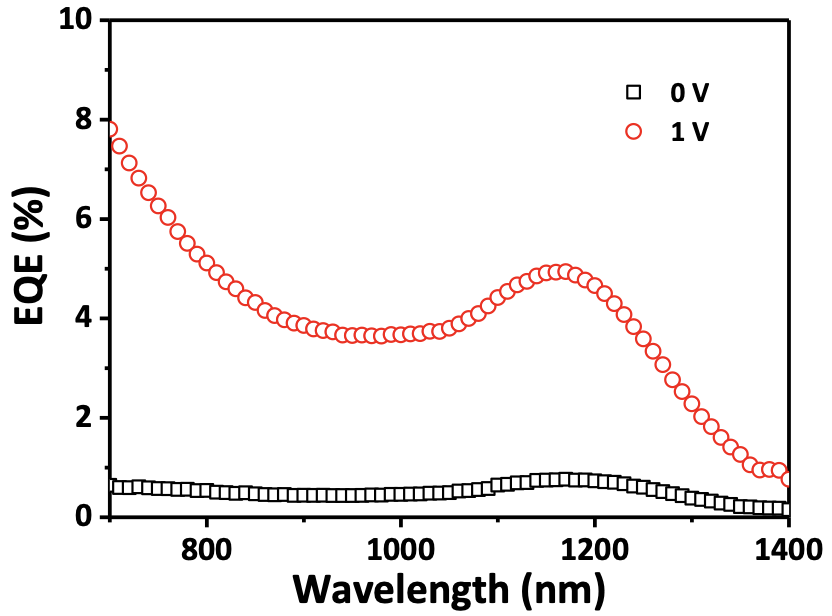


**Figure S11.** External quantum efficiency (EQE) spectra of precipitate InSb/InP CQDs photodetector biased from 0-1V (reverse bias).


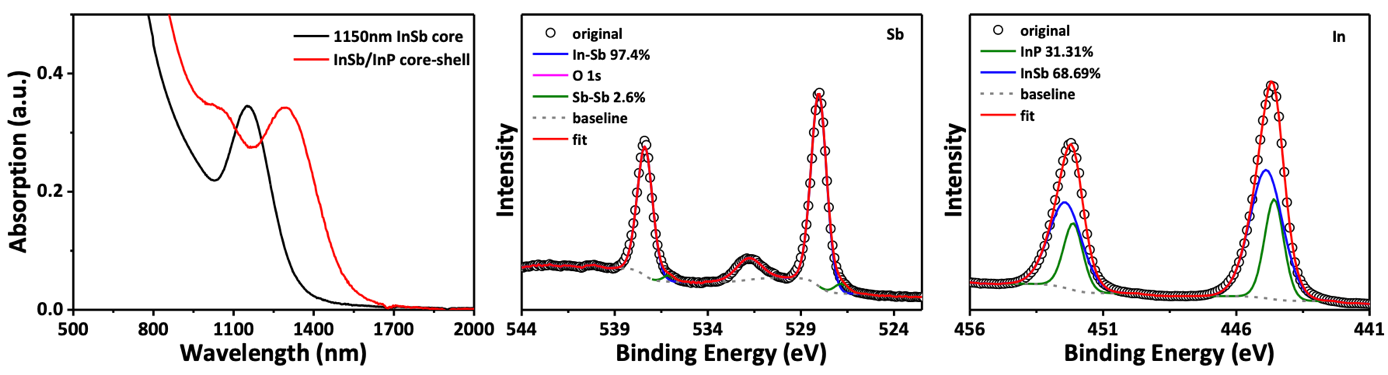


**Figure S12.** Absorption spectra of the larger size supernatant InSb CQDs before and after the InP shell growth (left). The In and Sb X-ray photoelectron spectroscopy (XPS) analysis of the corresponding films (right).


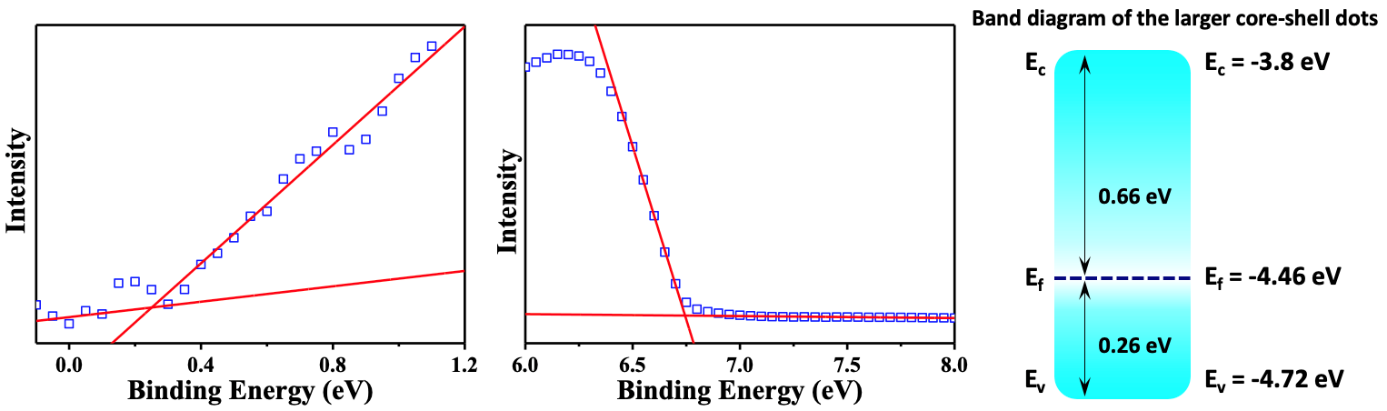


**Figure S13.** The energy level of the larger size supernatant InSb/InP core-shell CQDs films analyzed by the UPS spectra after InI_3_ ligand exchange.


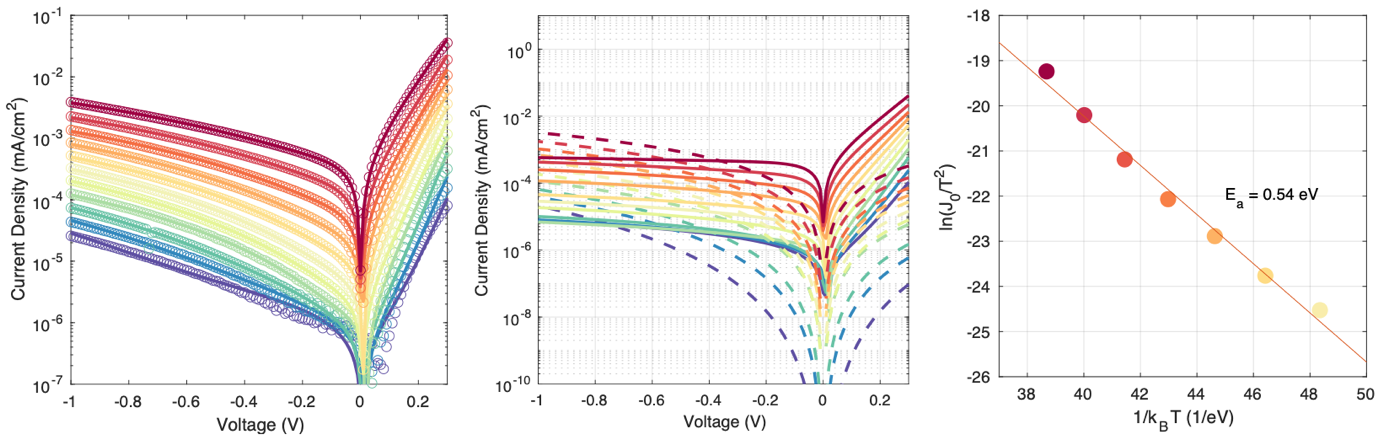


**Figure S14.** Diode analysis of supernatant InSb/InP core-shell CQDs devices. Left : Experimental *JV* curves (symbols) and their fittings (solid lines). Middle: Diode *JV* (solid lines) and trap assisted injection currents (dash lines). Right: Fitting of reverse saturation dark current density with thermal generation model.


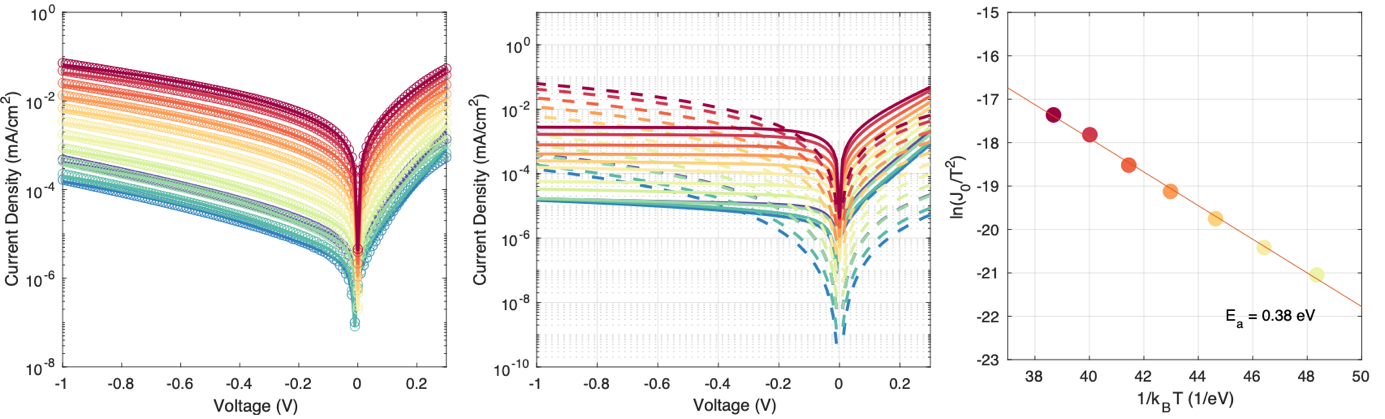


**Figure S15.** Diode analysis of precipitate InSb/InP core-shell CQDs devices. Left : Experimental *JV* curves (symbols) and their fittings (solid lines). Middle: Diode *JV* (solid lines) and trap assisted injection currents (dash lines). Right: Fitting of reverse saturation dark current density with thermal generation model.


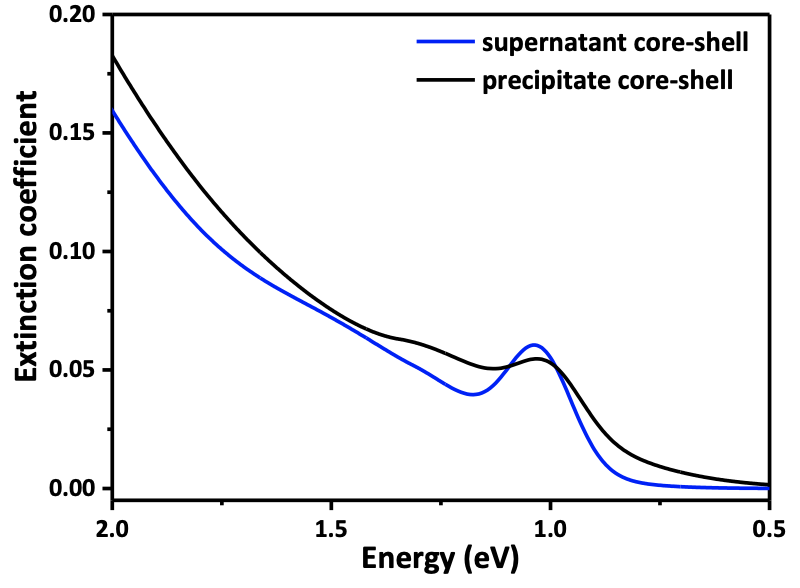


**Figure S16.** The extinction coefficients of supernatant and precipitate InSb/InP core-shell CQDs films. It is clear that the precipitate InSb/InP core-shell CQDs film shows lower band edge.


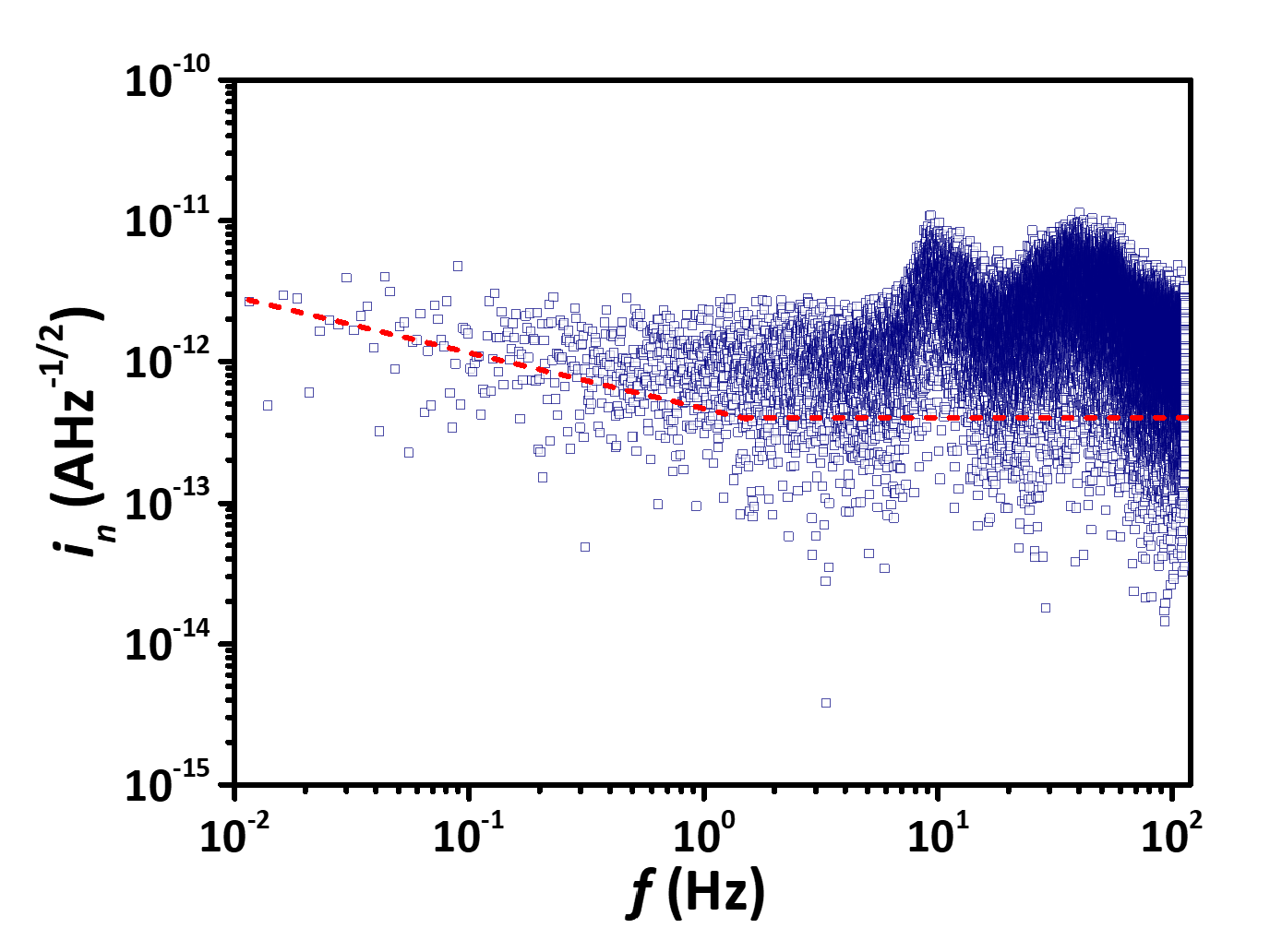


**Figure S17.** Frequency dependent noise spectral density measurement of InSb/InP core-shell CQDs photodetector at zero bias. It is measured by transient-current fast Fourier-Transform (FFT) method.


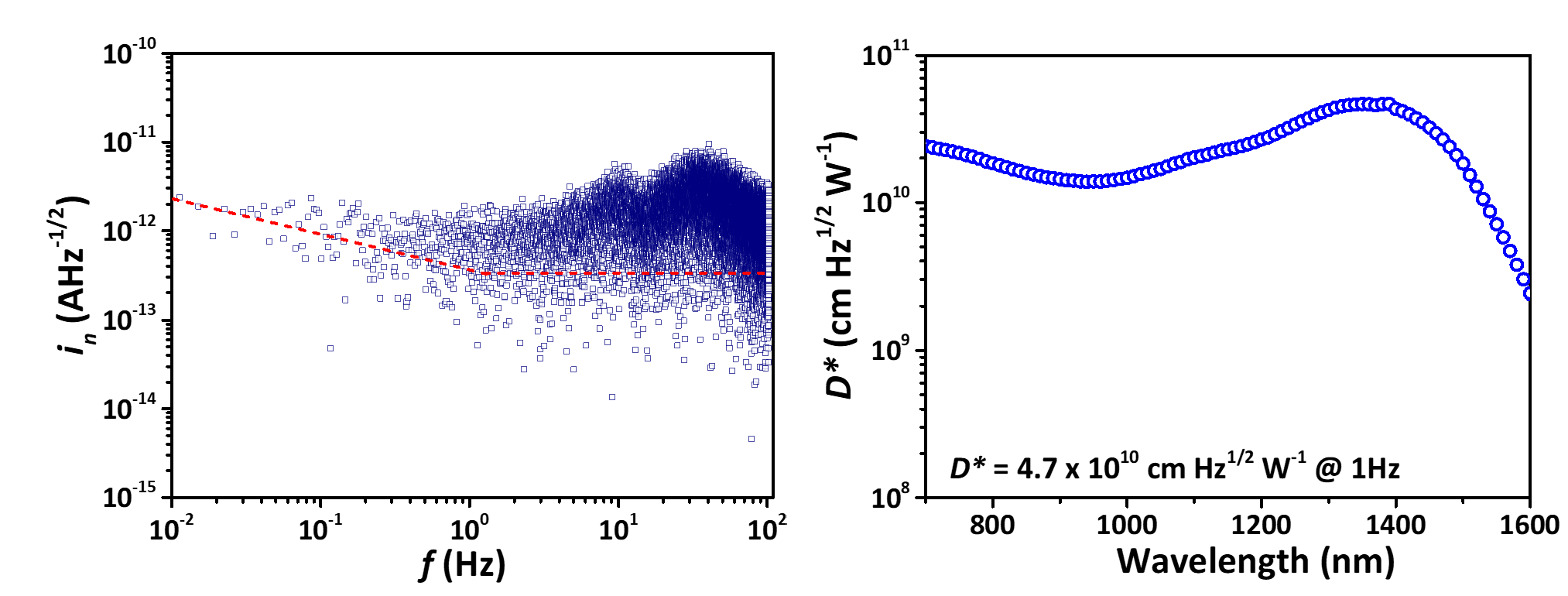


**Figure S18.** Frequency dependent noise spectral density measurement of larger size InSb/InP core-shell CQDs photodetector at zero bias (left). Specific detectivity spectrum of larger size InSb/InP core-shell QDs photodetector at 1 Hz according to the noise spectrum.


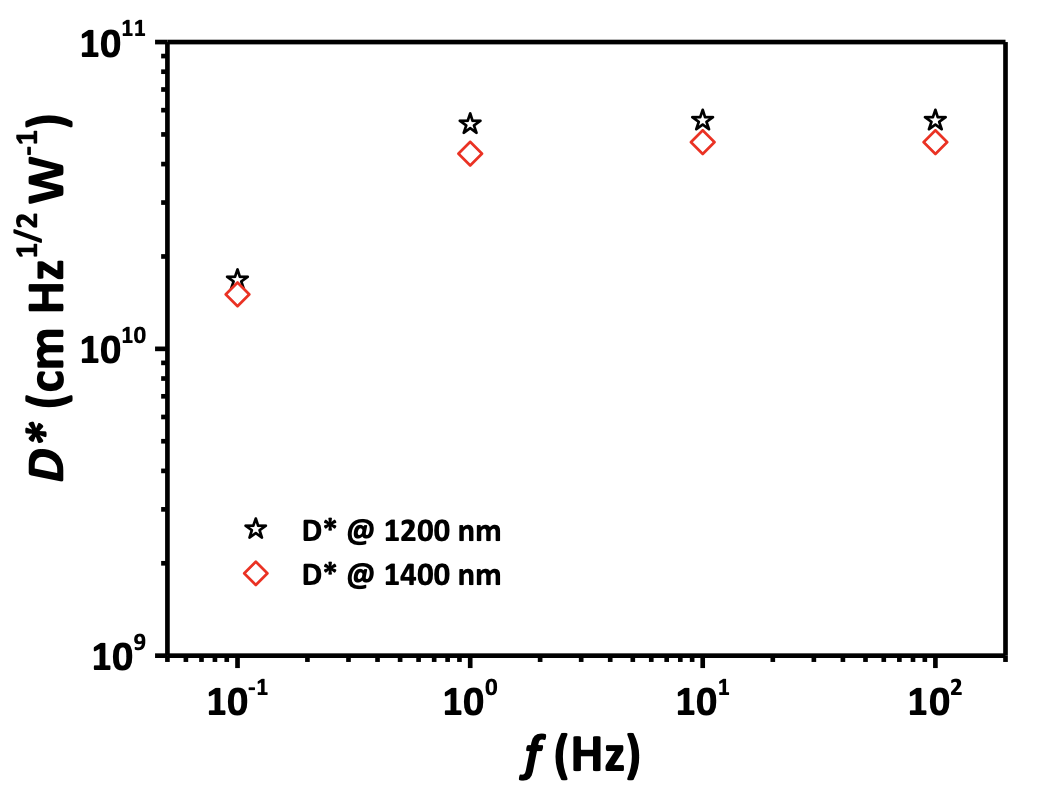


**Figure S19.** Frequency dependent specific detectivity (D*) of InSb/InP CQDs photodetector in this work.


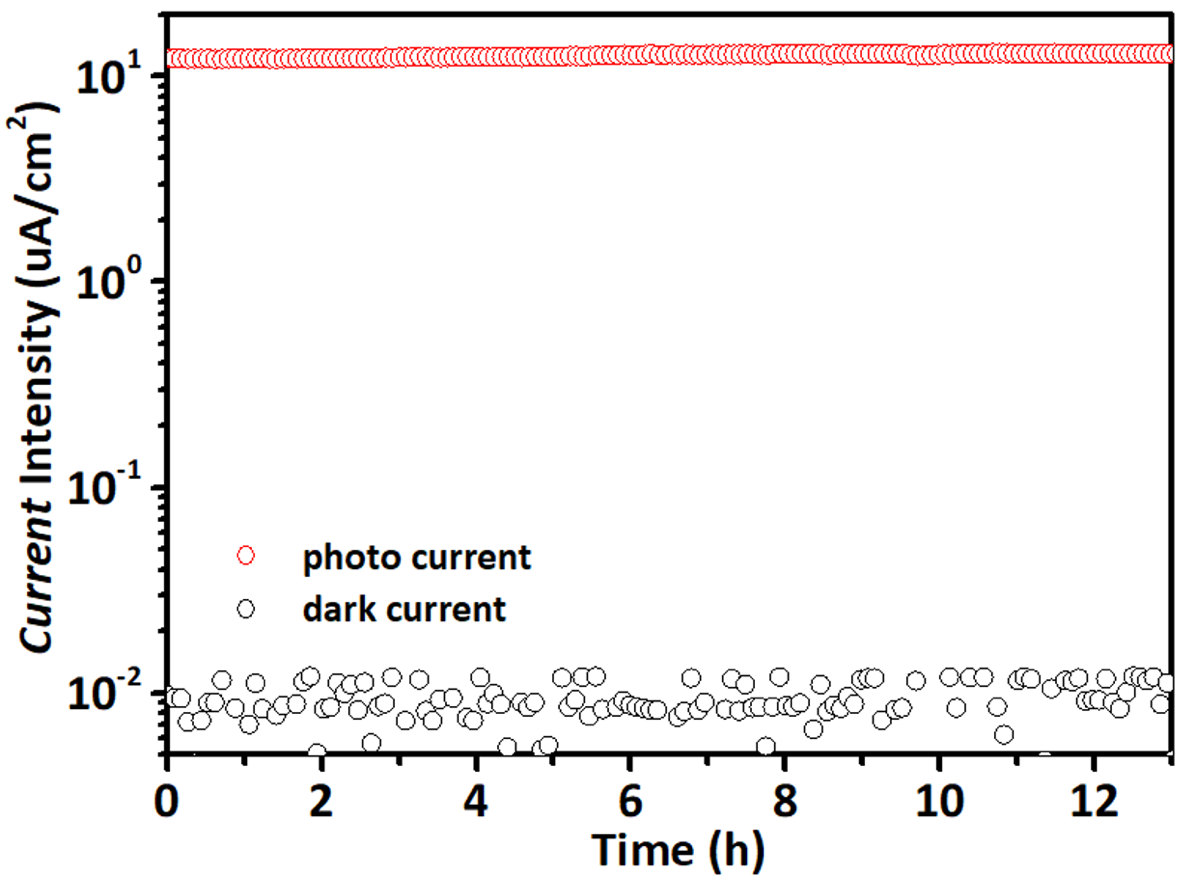


**Figure S20.** The operational stability of supernatant InSb/InP CQD photodetector with measured dark current and photo current.


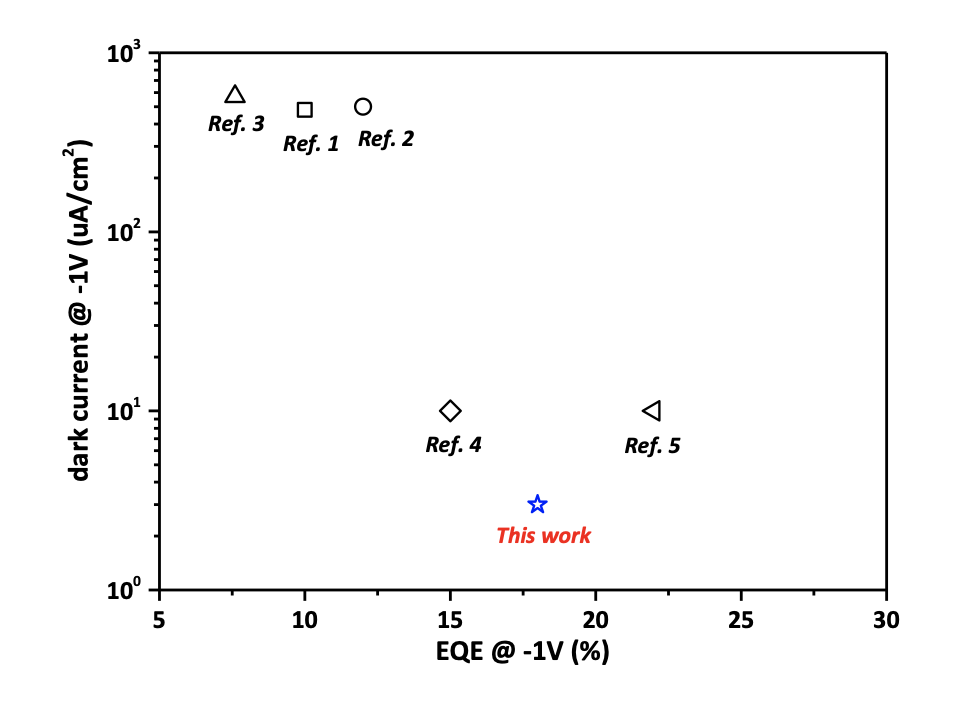


**Figure S21.** The EQE and *J_dark_* achieved in this work is compared to previously reported values of other III-V QDs photodetectors that are fabricated by the commercial non-pyrophoric precursors in the wavelength of 1350 nm which is highly relevant for imaging applications.

References

[1] M. Imran, D. Choi, D. H. Parmar, B. Rehl, Y. Zhang, O. Atan, G. Kim, P. Xia, J. M. Pina, M. Li, Y. Liu, O. Voznyy, S. Hoogland, E. H. Sargent. Halide-Driven Synthetic Control of InSb Colloidal Quantum Dots Enables Short-Wave Infrared Photodetectors. *Adv. Mater.* **2023**, *35*, 2306147.

[2] L. Peng, Y. Wang, Y. Ren, Z. Wang, P. Cao, G. Konstantatos. InSb/InP Core−Shell Colloidal Quantum Dots for Sensitive and Fast Short-Wave Infrared Photodetectors. *ACS Nano.* **2024**, *18*, 5113.

[3] M. S. Skorotetcky, W. J. Mir, T. Sheikh, K. E. Yorov, B. M. Saidzhonov, S. Daws, R. Zhou, M. N. Hedhili, M. Abulikemu, O. F. Mohammed, O. M. Bakr. Si-H Hydrosilane Reducing Agents for Size- and Shape- Controlled InAs Colloidal Quantum Dots. *Adv. Mater.* **2024**, *36*, 2412105.

[4] T. Sheikh, W. J. Mir, A. Alofi, M. Skoroterski, R. Zhou, S. Nematulloev, M. N. Hedhili, M. B. Hassine, M. S. Khan, K. E. Yorov, B. E. Hasanov, H. Liao, Y. Yang, A. Shamim, M. Abulikemu, O. F. Mohammed, O. M. Bakr. Surface-Reconstructed InAs Colloidal Nanorod Quantum Dots for Efficient Deep-Shortwave Infrared Emission and Photodetection. *J. Am. Chem. Soc.* **2024**, *146*, 29094.

[5] Y. Zhang, P. Xia, B. Rehl, D. H. Parmar, D. Choi, M. Imran, Y. Chen, Y. Liu, M. Vafaie, C. Li, O. Atan, J. M. Pina, W. Paritmongkol, L. Levina, O. Voznyy, S. Hoogland, E. H. Sargent. Dicarboxylic Acid-Assisted Surface Oxide Removal and Passivation of Indium Antimonide Colloidal Quantum Dots for Short-Wave Infrared Photodetectors. *Angew. Chem. Int. Ed.* **2024**, *63*, e202316733.
